# Geographical and temporal origins of *Neocaridina* species (Decapoda: Caridea: Atyidae) in Taiwan

**DOI:** 10.1186/s12863-019-0788-y

**Published:** 2019-11-21

**Authors:** Chiao-Chuan Han, Kui-Ching Hsu, Lee-Shing Fang, I-Ming Cheng, Hung-Du Lin

**Affiliations:** 1 National Museum of Marine Biology and Aquarium, Pingtung, 944 Taiwan; 2 Graduate Institute of Marine Biology, National Dong Hwa University, Pingtung, 944 Taiwan; 3 College of Fisheries, Guangdong Ocean University, Zhanjiang, 524088 China; 4 Center for Environmental Toxin and Emerging-Contaminant Research,Cheng Shiu University, Kaohsiung, 83347 Taiwan; 5 Department of Leisure and Sport Management, Cheng Shiu University, Kaohsiung, 83347 Taiwan; 6 Department of Marine Biotechnology and Resources, National Sun Yat-sen University, Kaohsiung, 80424 Taiwan; 7 The Affiliated School of National Tainan First Senior High School, Tainan, 701 Taiwan

**Keywords:** DNA barcoding, *Neocaridina*, Origin, Phylogeography, Approximate Bayesian computation

## Abstract

**Background:**

The freshwater species on Taiwan Island have been documented to have originated from mainland China and the Japanese islands from multiple events and by multiple colonization routes. Moreover, the sequences from the mitochondrial DNA cytochrome c oxidase subunit I (COI) have been used for DNA barcoding to identify the species. This study used the COI sequences to identify *Neocaridina* species in Taiwan and to examine their geographical and temporal origins.

**Results:**

In total, 479 specimens were collected from 35 localities, which covered almost all rivers in Taiwan. In addition, some sequences were downloaded from GenBank. The maximum likelihood (ML) tree displayed that all sequences were sorted into 13 taxa (clades), and all sequences in Taiwan were sorted into four clades. The Bayesian skyline plots revealed that these four *Neocaridina* species have declined recently in Taiwan.

**Conclusions:**

All results support that (1) there are four *Neocaridina* species in Taiwan, which are *N. davidi*, *N. saccam*, *N. ketagalan* and an undescribed *Neocaridina* species (*N.* sp.); (2) these four species colonized Taiwan Island in four colonization events; (3) *N.* sp. colonized Taiwan first; (4) after the island reached its shape, *N. ketagalan* and *N. saccam* colonized Taiwan from the Japanese islands and mainland China, respectively; (5) *N. davidi* colonized northern Taiwan last; and (6) the cyclic glacial and landform changes in East Asia shaped the colonization events and population structures of the *Neocaridina* species.

## Background

The genus *Neocaridina* Kubo, 1938, is a group of land-locked species of the family Atyidae that consists of 26 species and is distributed in East Asia [1, 2]. Based on the Taiwanese atyid shrimp fauna [3, 4], only one species, *N. denticulate*, is distributed throughout Taiwan Island. Shih and Cai [5] proposed two new species, *N. saccam* and *N. ketegalan*, in southern and northern Taiwan, respectively. However, Shin and Cai [5] only sampled the specimens of *N. ketegalan* in one population and that of *N. saccam* in the two populations. Thus, our study aimed to determine how many *Neocaridina* species are present in Taiwan as well as the distribution pattern of each species.

Taiwan Island is located off the southeastern coast of mainland China and is separated from China by the shallow Taiwan Strait. Taiwan was first isolated from the mainland by rising sea levels four to five million years ago (mya) and reached its present shape ca. 2 mya [6]. Previous biogeographic studies support that the many freshwater species easily migrated from the mainland to the island during the Pliocene and Pleistocene glaciations as a result of the lowered sea level [7–10]. Geological evidence indicates that during the glaciations, the land bridges connected Taiwan Island to the Asian continent three to four times, initially during the Pliocene glaciation and potentially two to three times during the Pleistocene glaciation [11, 12]. In addition, during the early and late Pleistocene, the sea basins of East Asia were exposed and Korea, the Japanese islands, Taiwan Island and mainland China connected [13, 14]. During these ice ages, the migrations between the Asian mainland and Taiwan, Ryuku Archipelago, and Japan may have been possible across the land bridges [15, 16]. Previous phylogeographical studies [9, 17, 18] have suggested that freshwater species in Taiwan might have originated from mainland China and Japan through multiple events and by multiple colonization routes.

**Fig. 1:**
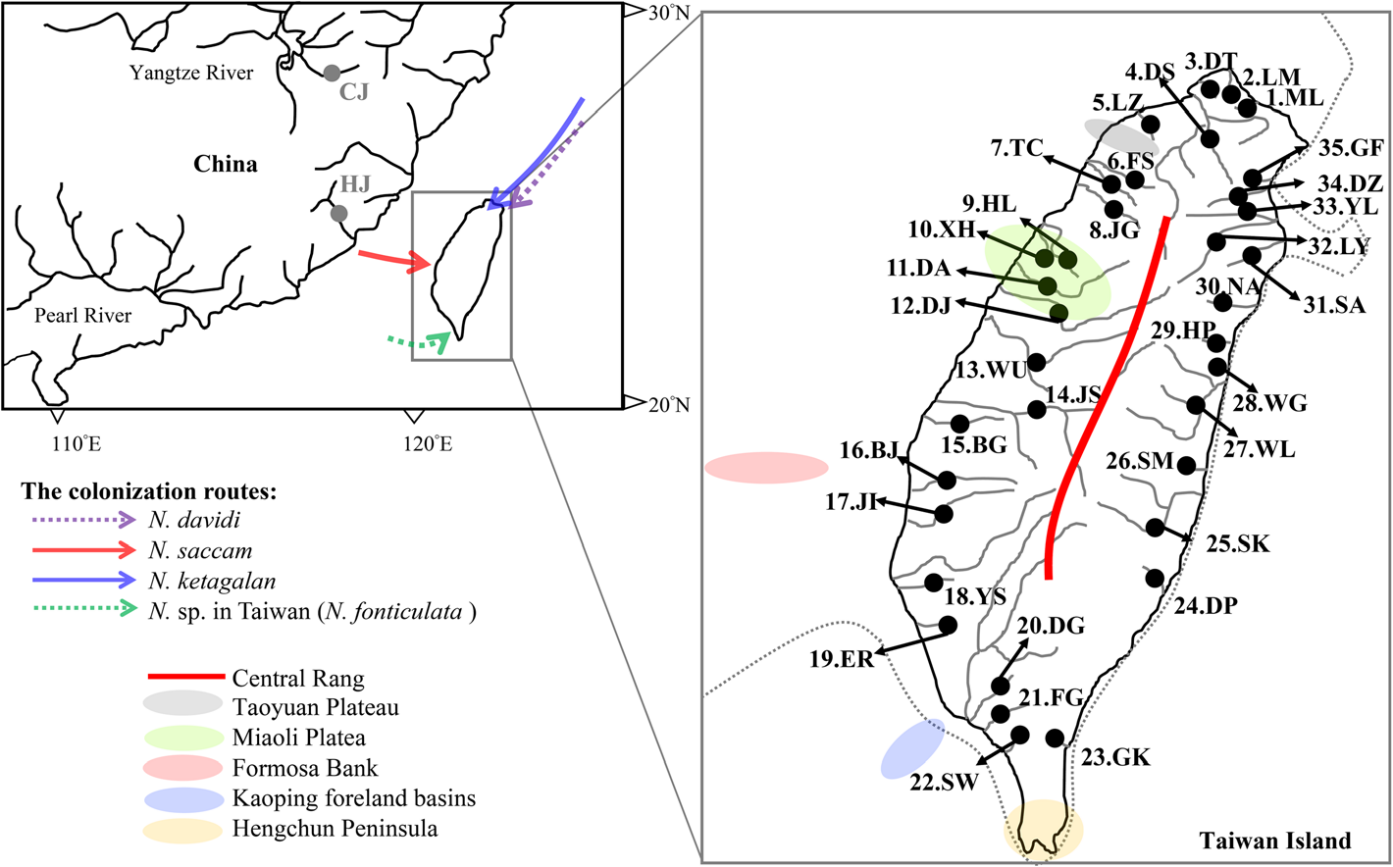
The sampling localities of the *Neocaridina* species in Taiwan are indicated by •. Possible colonization routes are displayed by arrows

Previous phylogeographic studies [18–20] have suggested that many geological barriers, e.g., the Central Range, Miaoli Plateau, and Kaoping foreland basins, shaped the structures and distribution patterns of the fauna in Taiwan Island. Taiwanese orogeny (mountain building) uplifted the longitudinal Central Range to nearly 4000 m (Fig. 1). The distribution patterns and phylogeographic studies of freshwater fishes [10, 19] indicate that the Central Range may have acted as a barrier to dispersal between the western and eastern populations. Lin [21] proposed that the Miaoli Plateau emerged at 0.150 mya based on geological studies, and many studies suggest that the Miaoli Plateau isolated freshwater fishes, preventing dispersal [9, 19]. The Kaoping foreland basins located in the southeastern Taiwan Strait reached a depth of 200 m within 3 km of the shoreline [22]. Previous studies [23–25] have proposed that this sea trench interrupted the extension of the Kaoping River towards the land bridge during the glaciations. In other words, even during the ice ages, the freshwater species south of the Kaoping River could not cross the Kaoping foreland basins to the north of the land bridge (Fig. 1).

According to geological history [11–14] and phylogeographic studies [9, 17, 18, 26], our study found that freshwater species colonized Taiwan Island from the Japanese islands or mainland China, once or multiple times, and through one or multiple routes. Moreover, the distribution patterns of the freshwater species were contributed by the origins of the colonization events and the colonization routes and times [9, 19]. Thus, our study also wanted to determine the distribution range of each *Neocaridina* species and their origins and phylogeographic patterns in Taiwan. To address the above problems, the mitochondrial DNA (mtDNA) cytochrome c oxidase subunit I (COI) was used to investigate the genetic diversity and structure of the *Neocaridina* species in Taiwan. The sequences of mtDNA are usually analysed in studies of animal phylogeography [9, 23, 27]. Among all the mtDNA genes, the COI gene is a widely accepted marker for resolving taxonomic identity and evaluating the levels of genetic diversity and differentiation in Decapoda species [28, 29]. The major questions in our study are (1) how many *Neocaridina* species are there in Taiwan, and (2) what is the colonization history of the *Neocaridina* species in Taiwan?

## Results

### Species diversity in Taiwan

**Fig. 2:**
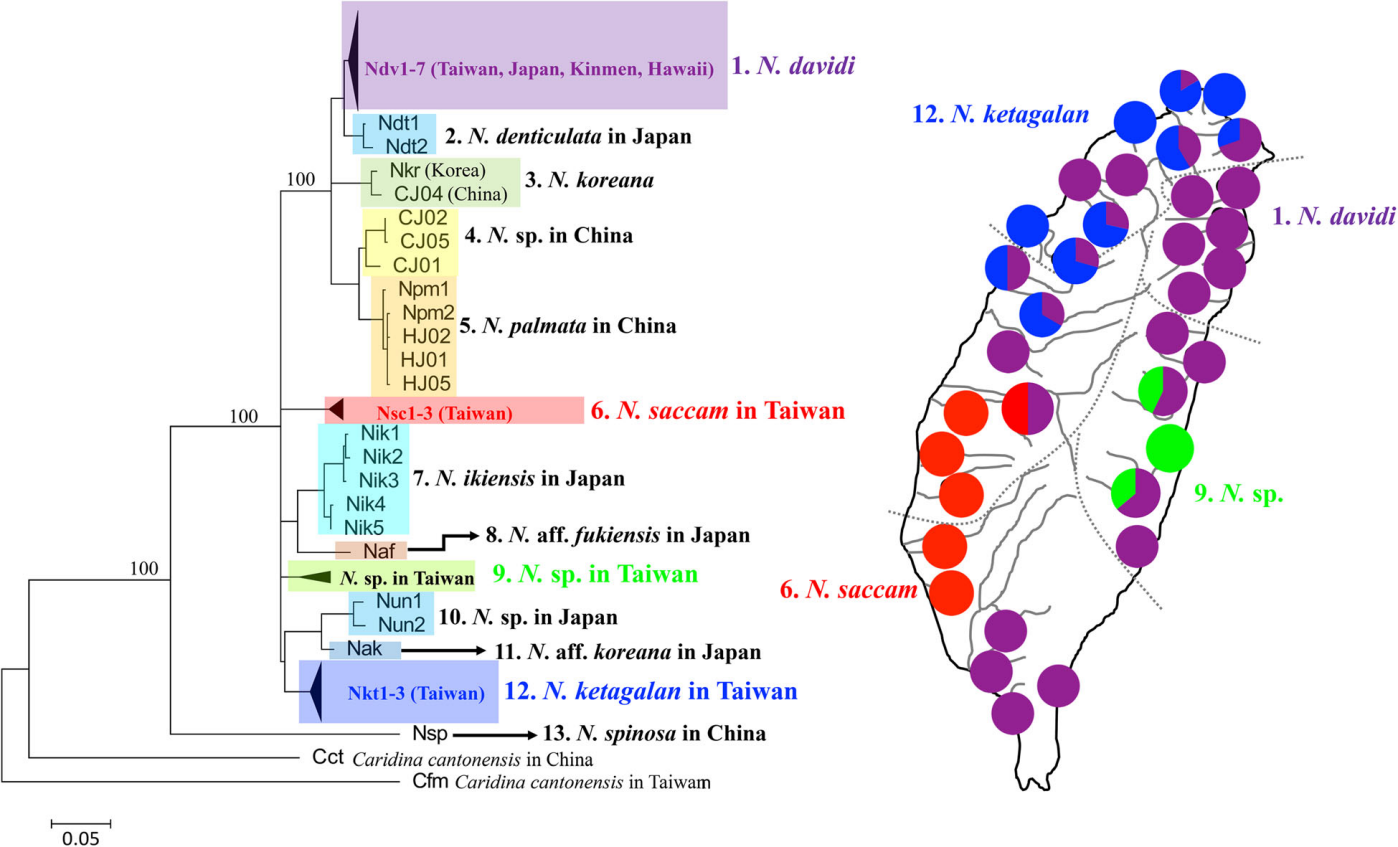
The ML tree of the mtDNA haplotypes in the *Neocaridina* species in Taiwan, China and Japan. The numbers at the nodes are bootstrap values

Based on all COI sequence data in our study (Table 1) and Shih et al. [5] from GenBank, the ML tree (Fig. 2) displayed that all sequences were assorted into 13 taxa (clades), and all sequences in Taiwan were assorted into four clades. The sequences of *N. davidi* in Taiwan, Kineme, Japan and Hawaii were grouped together and were close to those of *N. denticulata* in Japan. The sequences from the mainland (populations CJ and HJ) were assorted into three species [*N. koreana*, *N. palmata*, and one undescribed species (*N.* sp. in China)]. The range of the pairwise genetic distance between these 13 clades of *Neocaridina* (Fig. 2) was from 2.87% (between *N. davidi* and *N. denticulata*) to 15.23% (between *N.* sp. in Japan and *N. spinose*) (Table 2). The average pairwise genetic distance was 8.19%. These results suggested that there were four *Neocaridina* species in Taiwan: *N. davidi* (clade 1), *N. saccam* (clade 6), *N. ketagalan* (clade 12) and one undescribed species (*N.* sp. in Taiwan; clade 9). *Neocaridina davidi* was distributed widely; *N. saccam* was distributed in central and southern Taiwan; *N. ketagalan* was distributed in northern and southern Taiwan; and *N.* sp. was only distributed in eastern Taiwan (Fig. 2).

**Table 1:** Samples used for mtDNA analysis, species, location, code and summary statistics. The number of the private haplotypes (P) and the distribution information of shared haplotypes (S). For sample site number see Fig. 1

Species/ Population	Code	Sample size	Haplotype diversity (h)	P	S	Nucleotide diversity	
						π (%)	θ (%)
*N. davidi*		263	0.88			0.75	1.47
Malain	1.ML	16	0.23	2	–	0.04	0.05
Datun	3.DT	2	1.00	2	–	1.40	1.40
Danshuei	4.DS	23	0.61	2	D4, D10	0.27	0.30
Fongshan	6.FS	6	0.73	1	D7, D9	0.42	0.34
Touqian	7.TC	12	0.44	2	D7	0.07	0.10
Jhonggang	8.JG	1	–	0	D7	–	–
Houlong	9.HL	5	0.40	0	D2, D9	0.13	0.15
Daan	11.DA	15	0.83	2	D2, D3, D9	0.38	0.34
Dajia	12.DJ	6	0.80	4	–	0.26	0.34
Wu	13.WU	6	0.33	1	D9	0.05	0.07
Jhuoshuei	14.JS	15	0.34	0	D3, D9	0.05	0.05
Donggang	20.DG	6	–	0	D1	–	–
Fengkong	21.FG	18	0.58	2	D10	0.25	0.32
Shiwen	22.SW	1	–	0	D6	–	–
Gangkong	23.GK	11	0.44	0	D1, D6	0.07	0.05
Dapo	24.DP	12	0.17	0	D8, D9	0.03	0.05
Siuguluan	25.SK	23	0.52	0	D8, D9	0.08	0.04
Hualien	27.WL	24	0.16	0	D8, D9	0.03	0.04
Hualiengang	28.WG	12	–	0	D9	–	–
Heping	29.HP	2	–	0	D5	–	–
Naoao	30.NA	6	0.60	0	D5, D11	0.19	0.14
Suao	31.SA	5	0.40	2	–	0.19	0.23
Lanyang	32.LY	6	0.60	1	D4	0.09	0.07
Yilan	33.YL	18	0.87	6	D10, D11	0.60	0.77
Dezi	34.DZ	6	0.60	3	–	0.24	0.27
Gengfang	35.GF	6	0.73	3	–	0.31	0.27
*N. sp.*		43	0.64			0.33	1.12
Siuguluan	25.SK	13	0.53	3	N1	0.78	1.46
Sihmen	26.SM	12	–	1	–	–	–
Hualien	27.WL	18	0.37	1	N1	0.06	0.05
*N. saccam*		47	0.81			0.91	0.64
Jhuoshuei	14.JS	15	0.68	3	S2	0.16	0.14
Beigang	15.BG	3	–	0	S2	–	–
Bajhang	16.BJ	6	–	0	S1	–	–
Jishuei	17.JI	6	–	0	S1	–	–
Yanshuei	18.YS	1	–	0	S3	–	–
Erren	19.ER	16	0.34	2	S3	0.13	0.19
*N. ketagalan*		126	0.95			0.99	1.30
Malain	1.ML	7	0.29	2	–	0.49	0.70
Laomei	2.LM	7	0.71	2	K2	0.13	0.13
Datun	3.DT	10	0.53	4	–	0.16	0.28
Danshuei	4.DS	31	0.75	5	K2	0.52	0.47
Luzhou	5.LZ	12	–	1	–	–	–
Jhonggang	8.JG	6	0.53	2	–	0.08	0.06
Houlong	9.HL	12	0.53	1	K1	0.08	0.05
Xihu	10.XH	14	0.28	1	K3, K4	0.13	0.29
Daan	11.DA	15	0.60	2	K1, K3	0.32	0.24
Dajia	12.DJ	12	0.71	3	K4	0.23	0.26
total		479

**Table 2:** Matrix of the percentage of pairwise nucleotide divergences (p-distance) based on COI between clades of *Neocaridina* in Fig. 2

	2	3	4	5	6	7	8	9	10	11	12	13
1. *N. davidi*	2.87	4.90	5.08	4.96	7.08	7.18	8.04	7.50	9.10	7.52	6.68	13.00
2. *N. denticulata*		4.84	5.80	5.29	7.59	7.19	8.50	8.27	9.20	8.19	7.26	13.50
3. *N. koreana*			5.72	5.88	8.32	8.21	9.20	8.09	9.83	8.58	7.11	13.81
4. *N.* sp. (China)				3.49	7.23	8.28	9.46	8.04	9.67	8.42	7.70	13.29
5. *N. palmata*					7.98	8.05	8.86	8.22	9.92	8.64	7.17	14.13
6. *N. saccam*						7.28	8.31	6.91	7.82	6.51	5.74	13.36
7. *N. ikiensis*							5.62	6.36	6.61	6.55	5.95	13.94
8. *N.* aff. *Fukiensis*								6.99	7.80	7.18	6.64	14.44
9. *N.* sp. (Taiwan)									7.11	6.16	5.95	13.32
10. *N.* sp. (Japan)										3.74	7.19	15.23
11. *N*. aff. *Koreana*											5.49	14.13
12. *N. ketagalan*												13.70
13. *N. spinosa*												

### Population and demographic history

**Fig. 3:**
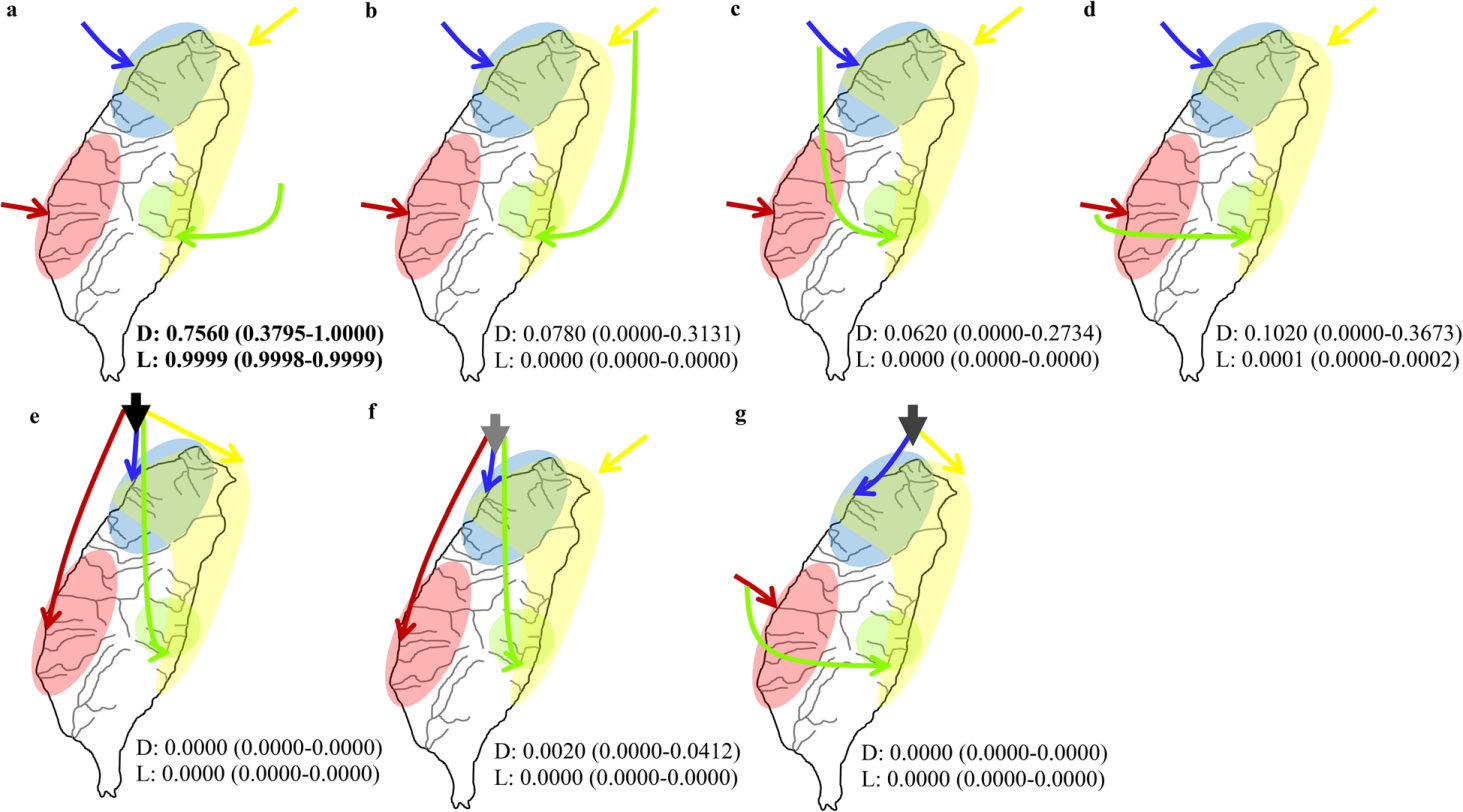
Graphical representation of the DIYABC analyses

Although the phylogeny of the *Neocaridina* species (Fig. 2) displayed that there were four species in Taiwan, this study attempted to understand the colonization routes of these four species. Thus, this study proposed seven population history scenarios with the program DIYABC to understand the colonization history of the *Neocaridina* species in Taiwan. In the first scenario (scenario A), which was based on the phylogenetic analysis (Fig. 2), these four species arrived in Taiwan via four different colonization events (Fig. 3a). Under scenarios B-D, according to a previous study [26], the freshwater fish in eastern Taiwan arrived from western Taiwan. Thus, we proposed that *N.* sp. might have originated from the other three species (Figs. 3b-d). Scenario E (Fig. 3e) showed that these four species colonized Taiwan by one colonization route and then diverged. In scenario F (Fig. 3f), *N. ketagalan*, *N. saccam* and *N. sp.* colonized Taiwan by the same colonization route and then diverged because these three species were allopatric. Finally, scenario G (Fig. 2g) showed that two species from northern Taiwan, *N. davidi* and *N. ketagalan*, colonized from one origin, and *N. saccam* and *N.* sp. colonized from another origin. The highest posterior probability was found for scenario A. Its posterior probability (D: 0.7560, 95% CI: 0.3795–1.0000; L: 0.9999, 95% CI: 0.9998–0.9999) was much higher than those of the other scenarios. The 95% CI of scenario A did not overlap with those for the other scenarios (Fig. 3). Thus, the *Neocaridina* species in Taiwan might have colonized the area through four different events.

**Fig. 4:**
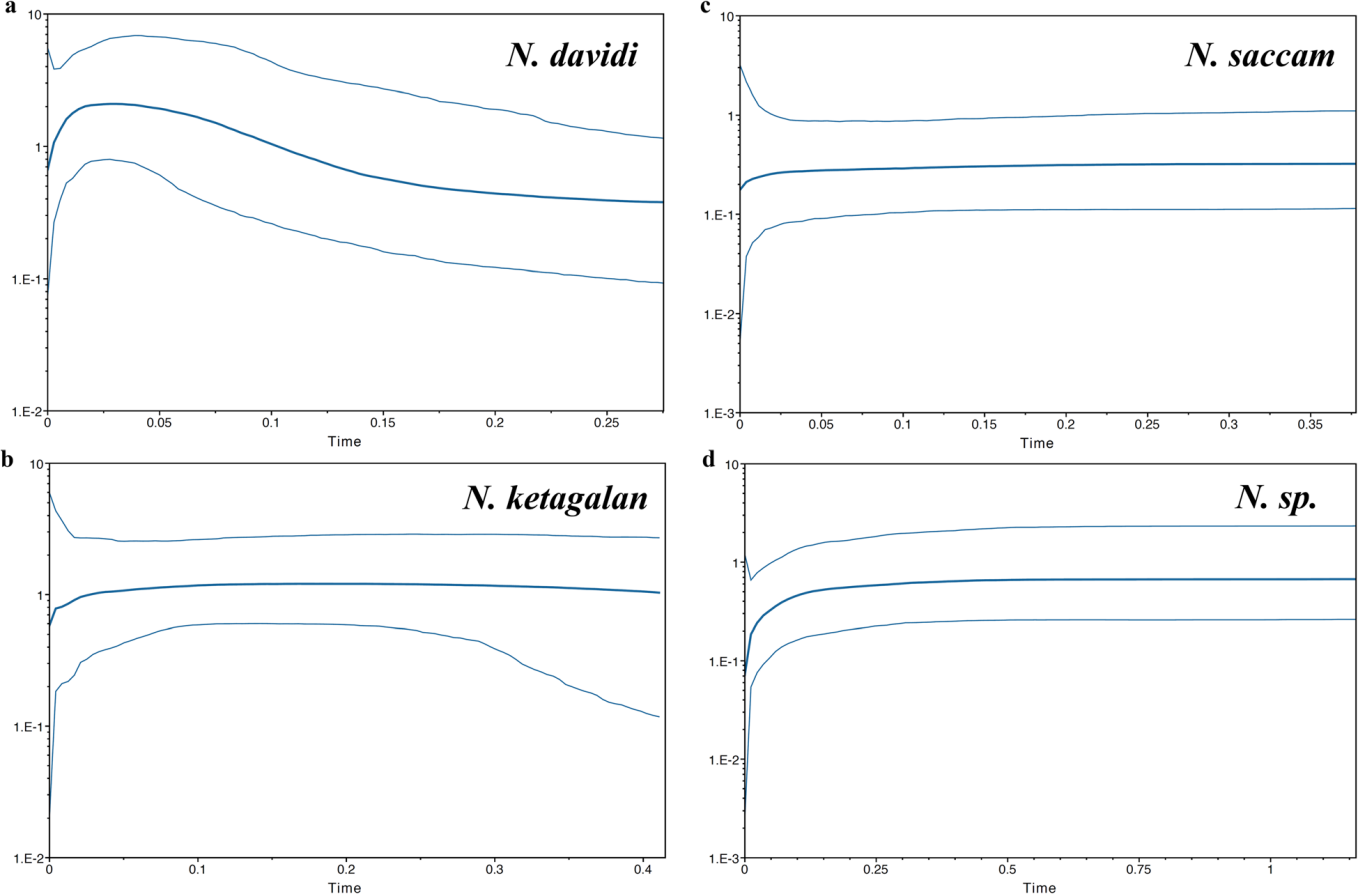
Bayesian skyline plot of the effective population sizes over time for *N. davidi* **a***, N. ketagalan* **b**, *N. saccam* **c** and *N.* sp. **d** in Taiwan

The time to coalescence was estimated in the BEAST analyses using two substitution rates: 2.33% per million years [2, 5, 30] and 1.1% per million years [31]. The T_MRCA_ of these four species, *N. davidi*, *N. ketagalan*, *N. saccam* and *N.* sp., were 0.242–0.532, 0.37–0.774, 0.432–0.938 and 1.021–2.180, respectively (Table 3). However, the estimated Tajima’s D and Fu’s Fs values were largely consistent within each species; excluding *N.* sp. (Table 3), none of the calculated values supported population expansion. Although the statistical analysis of the species-specific mismatch distributions (the SSD and Rg indices) revealed that the observed distributions were not significantly different from those expected under a sudden expansion model for all species (Table 3), the Bayesian skyline plots revealed that these four *Neocaridina* species declined recently in Taiwan (Fig. 4).

**Table 3:** Results of the dynamic tests and molecular clock analyses for the *Neocaridina* species in Taiwan.

Species	Tajima’s D	Fu’s *F*s	SSD	Rg	T_MRCA_	*N*_ST_	*G*_ST_
*N. davidi*	−1.443 (> 0.10)	−17.477 (0.000)	0.005 (0.840)	0.007 (0.950)	0.242 (0.125–0.374)	0.72	0.47
					0.532 (0.275–0.844)		
*N. ketagalan*	−0.727 (> 0.10)	−3.281 (0.016)	0.004 (0.430)	0.014 (0.300)	0.370 (0.202–0.560)	0.82	0.44
					0.774 (0.415–1.176)		
*N. saccam*	1.370 (> 0.10)	4.327 (0.022)	0.092 (0.070)	0.099 (0.120)	0.432 (0.176–0.727)	0.95	0.58
					0.938 (0.378–1.610)		
*N.* sp.	−2.414 (< 0.01)	1.092 (0.181)	0.021 (0.060)	0.158 (0.020)	1.021 (0.537–1.591)	0.28	0.51
					2.180 (1.163–3.449)		

The Tajima’s D, Fu’s *F*_S_ and mismatch distributions indices (i.e., sum of squared deviations from the sudden expansion model, SSD, and raggedness index, Rg) are reported. The corresponding *P*-values are given in brackets

### Population diversity of *N. davidi* in Taiwan

A total of 44 *N. davidi* COI haplotypes (641 bp) from 263 sequences were defined by 53 variable sites and 35 phylogenetically informative sites. The nucleotide sequences were A + T rich (60.0%). The mean COI haplotype diversity in each population was 0.47 (range: 0.00 to 1.00) (Table 1). The estimates of the current (θ_π_) and historical (θ_ω_) genetic diversity of each population indicated that most populations showed a pattern of decline (θ_π_ < θ_ω_) (Table 1). A comparison of the fixation indices *N*_ST_ and *G*_ST_ revealed that *N*_ST_ was larger than *G*_ST_ (0.72 and 0.47, respectively; Table 3). This result suggested a very weak relationship between phylogeny and geography.

**Fig. 5:**
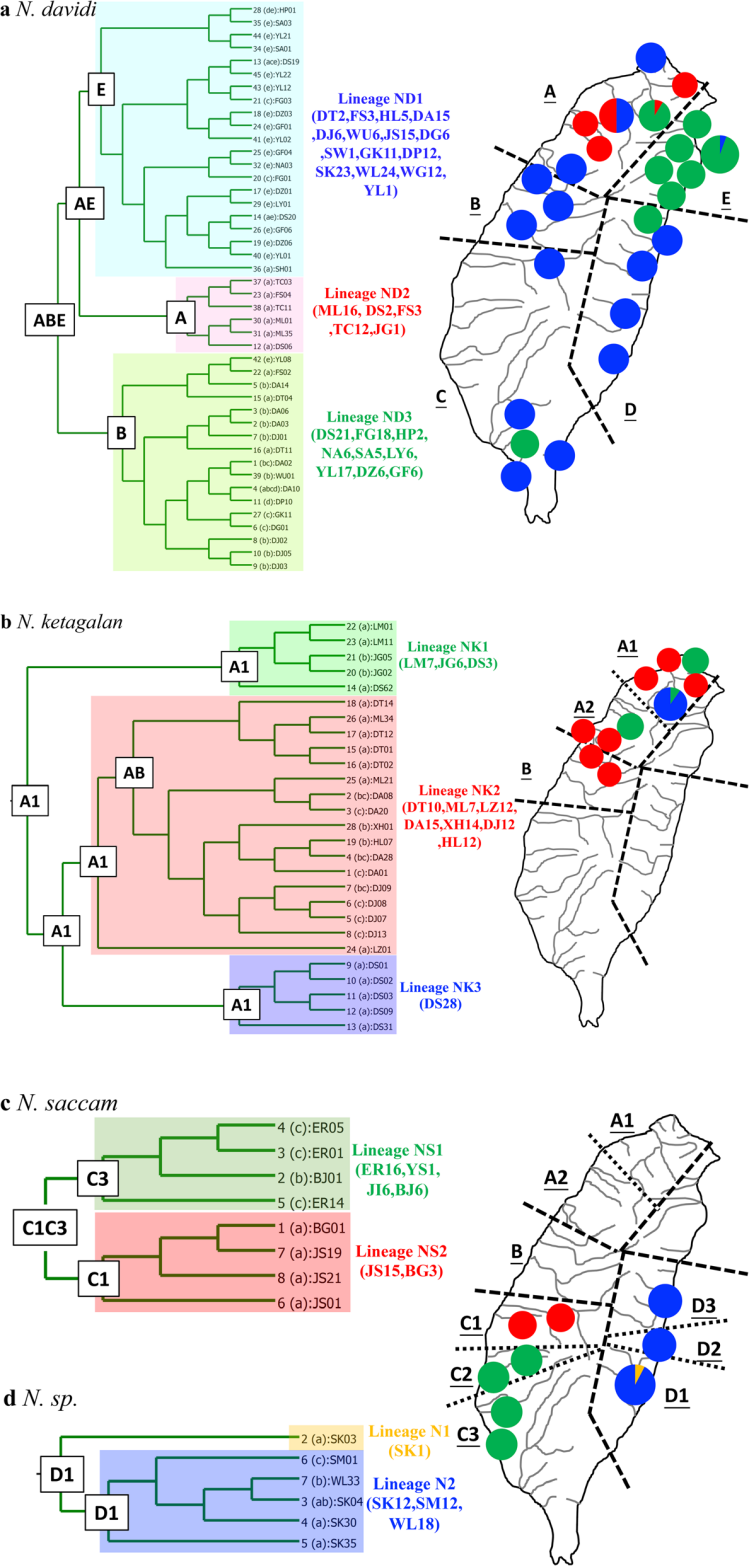
BEAST-derived chronograms of the mitochondrial DNA haplotypes of the *Neocaridina davidi* **a**, *N. ketagalan* **b**, *N. saccam* **c** and *Neocaridina* sp. **d** in Taiwan. The numbers at the nodes are the bootstrap values (maximum likelihood). The ancestral distribution inferred using S-DIVA is given in the box above each node. The frequencies of the lineages in each population and the range information are displayed on map

Among the 44 COI haplotypes, eleven haplotypes (D1-D11) were shared between two or more populations (Table 1). The most widespread haplotype was D9, which was distributed among nine populations. Among the 26 sampling populations, only one population (DA) had more than two shared haplotypes, and six populations (ML, DT, DJ, SA, DZ and GF) did not have any shared haplotypes. The population DA had the most shared haplotypes (D2, D3 and D9; Table 1). In the phylogenetic analyses, the haplotype trees reconstructed with different methods (ML and BI) were identical. In the BI tree (Fig. 5a), 44 mtDNA haplotypes fell into three lineages (ND1-ND3). Lineage ND1 included 15 populations that were widespread in Taiwan, lineage ND2 contained five populations in northern Taiwan, and lineage ND3 contained nine populations in northern, eastern and southern Taiwan (Fig. 5a).

To detect the ancestral region of *N. davidi* in Taiwan, all sampling populations were sorted into five regions as in previous studies: northern (A), central (B), southern (C), northeastern (E), and eastern (D) Taiwan [10, 32]. The results of the S-DIVA analysis produced a scenario with dispersion and vicariance events that shaped the current distribution patterns of *N. davidi* in Taiwan (Fig. 5a). The ancestral populations of *N. davidi* were distributed in northern, central and northeastern Taiwan and then diverged and dispersed widely in Taiwan.

### Population diversity of *N. ketagalan*

A total of 27 *N. ketagalan* haplotypes from 126 sequences were defined by 44 variable sites and 36 phylogenetically informative sites. The nucleotide sequences were A + T rich (58.2%). The mean COI haplotype diversity in each population was 0.49 (range: 0.00 to 0.75) (Table 1). The estimates of θ_π_ and θ_ω_ indicated that this species showed a pattern of decline (θ_π_ < θ_ω_) (Table 1). A comparison of the fixation indices *N*_ST_ and *G*_ST_ revealed that *N*_ST_ was larger than *G*_ST_ (0.82 and 0.44, respectively; Table 3). This result suggested a weak relationship between phylogeny and geography.

Among the 27 haplotypes, four haplotypes (K1-K4) were shared between two or more populations (Table 1). Among these ten sampling populations, two populations (XH and DA) had two shared haplotypes, and four populations (ML, DT, LZ and JG) did not have any shared haplotypes. The haplotype trees reconstructed with different methods (ML and BI) were identical. In the BI tree (Fig. 5b), 27 mtDNA haplotypes fell into three lineages (NK1-NK3). Lineage NK1 included three populations in northern Taiwan; lineage NK2 contained seven populations in northern and central Taiwan; lineage NK3 only contained one population (DS) in northern Taiwan (Fig. 5b). To detect the ancestral region, all sampling populations were sorted into three regions, A1, A2, and B (Fig. 5b). Northern Taiwan (A) was divided into two sub-regions (A1 and A2) by the Taoyuan Plateau (Fig. 1). The results of the S-DIVA analysis produced a scenario with vicariance and dispersal events that shaped the current distribution patterns (Fig. 5b). The ancestral populations of *N. ketagalan* were distributed north of the Taoyuan Plateau and then dispersed southward.

### Population diversity of *N. saccam* and *N.* species in eastern Taiwan

A total of eight *N. saccam* COI haplotypes from 47 sequences were defined by 17 variable sites and 15 phylogenetically informative sites. The nucleotide sequences were A + T rich (58.7%). The mean COI haplotype diversity in each population was 0.17 (range: 0.00 to 0.68) (Table 1). The haplotype and nucleotide diversities in most populations were 0.00. This result revealed very high levels of differentiation among the populations. A comparison of the fixation indices *N*_ST_ (0.95) and *G*_ST_ (0.58) revealed a weak relationship between phylogeny and geography (Table 3). Among the eight haplotypes, three haplotypes (S1-S3) were shared between two adjacent populations (Table 1). The haplotype trees reconstructed with different methods (ML and BI) were identical. In the BI tree (Fig. 5c), all haplotypes fell into two lineages (NS1 and NS2). Lineage NS1 included four populations south of the Formosa Bank, and lineage NS2 contained two populations north of the Formosa Bank (Figs. 1 and 5c). The results of the S-DIVA analysis showed that the ancestral populations of *N. saccam* were distributed in north- (C1) and south (C2) of Formosa Bank (Figs. 1 and 5c).

The six haplotypes from the 43 sequences of *N*. sp. in eastern Taiwan were defined by 30 variable sites and 6 phylogenetically informative sites. The nucleotide sequences were A + T rich (59.1%). The mean haplotype diversity in each population was 0.30 (range: 0.00 to 0.53) (Table 1). A comparison of the fixation indices *N*_ST_ (0.28) and *G*_ST_ (0.51) displayed that the most related haplotypes were found in different populations (Table 3). Compared with those of other species, the *N*_ST_ of *N*. sp. was the smallest (Table 3). These results suggested that the level of population differentiation of *N*. sp. was much lower than that of the other three species. The phylogenetic analyses also revealed mixed populations (Fig. 5d). The results of the S-DIVA analysis indicated that the ancestral population was distributed in the population SK and then to the north.

## Discussion

### Systematics of the genus *Neocaridina*

Many studies suggest that species should fulfil two criteria, monophyly and distinctness [33–35]. In the present study, the freshwater shrimp *Neocaridina* in Taiwan formed four monophyletic clades (clades 1, 6, 9 and 12; Fig. 2), and the mean genetic distance among these four clades was 6.64% (ranging 5.74 to 7.50%; Table 2). The range of the pairwise genetic distance between these 13 clades of *Neocaridina* (Fig. 2) was from 2.87% (between *N. davidi* and *N. denticulata*) to 15.23% (between *N.* sp. in Japan and *N. spinose*), and the average pairwise distance was 8.19% (Table 2). Robe et al. [29] evaluated the utility of mtDNA COI in the identification of species of Palaemonidae (Crustacea, Decapoda) and found that the mean genetic distances between the species within the genus *Macrobrachium* ranged from 0.000 to 0.312 (mean = 0.198). Hebert et al. [36] suggested that the best threshold for distinguishing intra- from interspecific divergence was approximately 3% sequence divergence, although this value was later modified approximately ten times by many studies [37–40]. Thus, the present study suggested that the four clades in Taiwan corresponded to four species: *N. davidi, N. saccam*, *N. ketagalan* and one undescribed species (*N.* sp. in Taiwan) (Fig. 2; Table 2). Our study provides a table comparing the morphologic characteristics among these four *Neocaridina* species in Taiwan and their identification keys (Table 4).

**Table 4:** Comparison of the morphological characters among four *Neocaridina* species in Taiwan and their identification keys

	*N. davidi*	*N. saccam*	*N. ketagalan*	*N.* sp.
1. The merus of the 1st pereiopod (as long as wide)	2.5	1.4–1.7	1.8–2.2	1.8–2.0
2. The carpus of the 1st pereiopod in the male (as long as high)	1.7	1.4	1.6	1.2–1.5
3. The number of accessory spines on the dactylus of the 3rd pereiopod	5–8	4–6	4–6	4–6
4. Endopod of the male 1st pleopod (as long as wide)	1.2	1.4	1.4	1.7
5. Propodus of the 3rd pereiopod (as long as dactylus)	3.0	2.7–3.2	3.5–3.9	2.7–3.0
6. Dactylus of the 5th pereiopod (as long as broad)		3.0–3.5	3.7–4.0	2.9–3.4
7. The number of spinules on the uropodal diaeresis	9–13	12–14	14–17	13–14
Keys to Taiwan species of the *Neocaridina* group:				
1a. Rostrum reaching slightly beyond the end of the 1st segment of the antennular peduncle, endopod of the male 1st pleopod 1.7 times as long as wide				*N.* sp.
1b. Rostrum reaching or more than the mid-length of the 2nd of antennular peduncle, endopod of the male 1st pleopod less than 1.7 times as long as wide				2
2a. Carpus of 1st pereiopod in the male more than 1.6 times as long as high				3
2b. Carpus of first pereiopod in the male 1.4 times as long as high				*N. saccam*
3a. Propodus of the 3rd pereiopod more than 3.5 times as long as dactylus, pleopod 1.3–1.5 times as long as wide, endopod of the male 1st pleopod less than 1.4 times as long as wide				*N. ketagalan*
3b. Propodus of the 3rd pereiopod less than 3.5 times as long as dactylus, endopod of the male 1st pleopod less than 1.2 times as long as wide				*N. davidi*

Recently, Shih et al. [41] proposed the third endemic species of *Neocaridina* known from Taiwan. This new species of land-locked freshwater shrimp, *N. fonticulata*, is described from Kenting, Hengchun Peninsula, and southern Taiwan [41]. However, although our study sampled specimens from 35 localities in Taiwan, which covers almost all rivers within the island, we did not sample any specimens in the Hengchun Peninsula (Fig. 1). After comparing the morphological characteristics (Table 4 and Shih et al. [41]), our study found that *N.* sp. in Taiwan may be synonymous with *N. fonticulata*. However, the molecular data of *N. fonticulata* have not been released. Therefore, this question needs to be confirmed in the future.

Moreover, our study found that the systematics of *N. davidi* were unclear. *Neocaridina davidi*, once named *N. denticulate sinensis* [5], and Shih et al. [2] suggested that it was synonymous with *N. davidi*. However, our study found that the genetic distance between *N. davidi* and *N. denticulata* was the smallest (2.87%). Thus, we could not suggest that “*N. davidi* in Taiwan” was a species, subspecies or population. The systematics and distribution area of *N. denticulata* were also unclear. Moreover, our study also found that there were many questions about the systematics of the genus *Neocaridina* in East Asia. For example, clade 11 (name: Nak; accession no. LC324777) was named *N. aff. Koreana* [2], but it is not close to *N. koreana* (clade 3; Fig. 2) (name: Nkr; accession no. LC324768 in Shih et al. [2]). Furthermore, undescribed *Neocaridina* species were found not only in our study (Fig. 2; clade 4, *N.* sp. in China, and clade 9, *N.* sp. in Taiwan) but also in the study of Shih et al. [2] (clade 10, *N.* sp. in Japan). Our study suggests that the systematics and species diversity of the genus *Neocaridina* in East Asia need revisions in future studies.

As described above, the diversity of the genus *Neocaridina* is outside of our understanding. The distribution of the shared haplotypes (Table 1) and *N*_ST_ (Table 3) within each species showed high population differentiation. These results suggested weak migrating potential in the *Neocaridina* species. Thus, their distribution patterns were restricted (Fig. 2), and the ancestral populations were easily isolated. These results were also supported by the DIYABC analysis (Fig. 3). The four species did not colonize Taiwan by one colonization route (scenario E, Fig. 3e) because the ancestral populations could not disperse over the entire island. Thus, these four *Neocaridina* species in Taiwan may have colonized the islands through four different groups of ancestral populations.

### Multiple origins of the genus *Neocaridina* in Taiwan

Our study found four *Neocaridina* species in Taiwan. The distribution ranges of the three species, *N. saccam*, *N. ketagalan* and *N.* sp., were restricted, and only *N. davidi* was widely distributed (Fig. 2). The phylogenetic analysis of *Neocaridina* species in the world revealed that these four species in Taiwan were polytomous (Fig. 2). The T_MRCA_ of these four Taiwanese species were different (Table 3). Moreover, the results of the DIYABC analysis demonstrated that these four *Neocaridina* species colonized Taiwan during four colonization events (Fig. 3). Chang et al. [19] also found that two endemic *Microphysogobio* species colonized Taiwan from two origins and through two colonization centres. Previous studies [9, 19, 42] propose that due to the geological history of Taiwan Island, the different colonization times shaped the different distribution patterns. These present results of the genus *Neocaridina* in Taiwan agreed with those of previous studies [9, 19, 42]. The four *Neocaridina* species in Taiwan displayed different distribution patterns, and they may have colonized the islands at different times.

Many studies [9, 18, 19, 42] have suggested that when the freshwater species colonized Taiwan after the island reached its present shape, their distribution range was restricted. Among the four species in Taiwan, *N.* sp. was restricted to eastern Taiwan. However, the distribution patterns of the freshwater fishes and the phylogeographic studies [10, 19] indicate that the Central Range has acted as a barrier to dispersal between the western and eastern populations of species. Thus, many freshwater species were not distributed in eastern Taiwan, and some species were distributed in eastern Taiwan by human activities [26]. Thus, the freshwater species in eastern Taiwan colonized before those in western Taiwan or originated from populations in western Taiwan through human activities. The results of T_MRCA_ estimated that *N.* sp. colonized before other species (Table 3). Based on the substitution rate of 1.1% per million years [31], the T_MRCA_ of *N.* sp. was 2.180 mya, which was before the Central Range in Taiwan formed (ca. 2 mya). In addition, the results of the DIYABC analysis also supported that the genus *Neocaridina* colonized Taiwan through four colonization events. Thus, this study suggested that *N.* sp. colonized Taiwan before the island reached its current shape, and the T_MRCA_ based on the substitution rate of 1.1% per million years was likely an appropriate estimate.

According to previous studies [9, 17, 18, 20, 26], the freshwater species colonized Taiwan through five colonization centres: two to the south of the Formosa Bank, two to the north of the Formosa Bank and the south of the Miaoli Plateau, and one to the north of the Miaoli Plateau. In the phylogeny of the genus *Neocaridina* (Fig. 2), *N. ketagalan* was grouped with *N.* aff. *Koreana* and *N.* sp. in Japan as monophyletic. The pairwise p-distance between the clades of *Neocaridina* suggested that *N. ketagalan* was close to *N.* aff. *Koreana* in Japan (Table 2). Moreover, the S-DIVA analyses showed that the ancestral populations of *N. ketagalan* were distributed north of the Taoyuan Plateau (Fig. 5b). Our study found that *M. brevirostris* and *N. ketagalan* had the same distribution area, but Chang et al. [19] proposed that *M. brevirostris* might have originated from mainland China. However, in a study by Chang et al. [19], we found that *M. brevirostris* was close to *M. koreensis* from South Korea. Moreover, Chiu et al. [18] found that the freshwater snail in northern Taiwan originated from Japan. Thus, this study considered that the freshwater species in northern Taiwan might not have colonized from mainland China and suggested that *N. ketagalan* originated from Japan (Fig. 1).

*Neocaridina saccam* was only distributed south of the Miaoli Plateau, and the results of the S-DIVA analysis demonstrated that the ancestral populations of *N. saccam* were distributed south and north of the Formosa Bank (Figs. 1 and 5c). Based on these results, *N. saccam* did not colonize Japan. According to the geographic locations, our study suggested that *N. saccam* originated from mainland China (Fig. 1). Actually, this colonization route was the most common to Taiwan Island [17, 23, 32, 43]. In addition, our study found that *N.* sp. in eastern Taiwan may be synonymous with *N. fonticulata* in the Hengchun Peninsula (Fig. 1). If this hypothesis is supported, *N.* sp. was also distributed in southern Taiwan. This distribution pattern was similar to that of two freshwater fishes, *Spinibarbus hollandi* and *Onychostoma alticorpus*. Chiang et al. [20] proposed that these two fishes colonized the island in the southern region of the Kaoping foreland basins, followed by eastern and northward dispersal. Thus, our study considered that *N.* sp. in Taiwan (*N. fonticulata*) may have colonized mainland China (Fig. 1).

In addition, although the S-DIVA analyses showed that the ancestral populations of *N. davidi* were distributed in northern Taiwan, this species is widely distributed around the world. Moreover, the phylogenetic analysis showed that *N. davidi* was close to *N. denticulata* in Japan. *Neocaridina davidi* may have colonized Japan (Fig. 1). However, our study could not suggest a geographical origin because the systematic status of this species was unidentified. Accordingly, this study suggested that the *Neocaridina* species in Taiwan colonized the area from multiple geographical and temporal origins, but the deterministic geographical sources need further study.

### Population history of *N.* sp. in eastern Taiwan

Our study found that *N.* sp. was distributed only in three adjacent rivers: SK, SM and WL (Figs. 2 and 5d). Although the *N*_ST_ in this species was smaller than those in other species, only this species displayed a higher *G*_ST_ than *N*_ST_ (Table 3). These results suggested that most related haplotypes were found in different populations. However, the depth of the sea around eastern Taiwan was deeper than the depth in western Taiwan (Taiwan Strait), and even during the glaciations, these oceans around eastern Taiwan were not exposed. Previous studies have demonstrated that the amphidromous fish *R. giurinus* [43] and shrimp *Caridina pseudodenticulata* [42] larvae survived in seawater and could not cross this deep sea. How did *N.* sp. colonize or migrate among these three rivers? The geological study of Taiwan Island [44] proposed that these three adjacent rivers belonged to one river, the paleo-Siuguluan River (paleo-SK), and separated after the middle Pleistocene.

### Phylogeography of *N. saccam* and *N. ketagalan*

*Neocaridina saccam* were divided into two lineages, NS1 and NS2 (Fig. 5c), and exhibited a southern and northern distribution, which were to the south and to the north of the Formosa Bank. The S-DIVA analysis showed that the ancestral populations of *N. saccam* were distributed to the south and north of Formosa Bank (Fig. 5c). Moreover, only two populations, JS, the northernmost population, and ER, the southernmost population, had private haplotypes (Table 1; Fig. 1). These results seemed to reveal that the colonization route was divided into two routes by the Formosa Bank. The Formosa Bank is located in the southern part of the Taiwan Strait. Previous studies [9, 17, 19, 45] have suggested that the Formosa Bank divided the glacial land bridge in the Taiwan Strait; however, the role of the Formosa Bank on the population dispersion within the island was rarely described. Ju et al. [26] proposed that during the maximum glacial periods, the ridge lifted from Formosa Bank to the present coastline of Taiwan Island. Therefore, during the maximum glaciation, the dispersal between the two sides of the bank through the exposed continental shelves of the island were prevented. After *N. saccam* colonized the island, the northward dispersal route was interrupted by the Miaoli Plateau, and the southward dispersal route was fragmented by the Kaoping foreland basins (Fig. 1).

*Neocaridina ketagalan* can be divided into three lineages (NK1-NK3, Fig. 5b). Lineage NK3 was restricted to the northern region of the Taoyuan Plateau, lineage NK1 was restricted to the north region of the Miaoli Plateau, and lineage NK2 was restricted to the north region of the Formosa Bank (Figs. 1 and 5b). The S-DIVA analysis displayed that its ancestral populations were distributed north of the Taoyuan Plateau and then southward (A1 region; Fig. 5b). Finally, the population structure was shaped by the Taoyuan Plateau, Miaoli Plateau and Formosa Bank. The Taoyuan Plateau is located in northwestern Taiwan (Fig. 1). Some freshwater fishes, e.g., *O. evolans*, *Squalidus argentatus*, *Sinibrama macrops* and *Hemibarbus labeo*, were only distributed in the Tamsui River north of the Taoyuan Plateau (excluding). Chang et al. [19] and Hsu et al. [23] also found that the Taoyuan Plateau divided the populations of *M. brevirostris* and *Semisulcospira libertina* into different lineages. Thus, the lineage NK3 was restricted to the northern region of the Taoyuan Plateau. Moreover, many studies suggest that the Miaoli Plateau prevented the dispersal of the freshwater fishes [9, 19]. Thus, when the Miaoli Plateau emerged, the populations were isolated and diverged (lineage NK1). Last, as described above, during the maximum glaciation, the Formosa Bank interrupted the migrations of *N. saccam* and *N. ketagalan*.

### Population history of *N. davidi* in Taiwan

Among the four *Neocaridina* species in Taiwan, the distribution range of *N. davidi* was wider than those of the others (Fig. 2). According to a previous study [9, 18], this widely distributed species colonized Taiwan Island before the species with restricted ranges. However, the results of the T_MRCA_ analysis showed that *N. davidi* colonized the islands after the other species (Table 3). In addition, our study also found that this species was widely distributed throughout the world. *Neocaridina davidi* is known to be an invasive species due to its importance in the aquarium trade (Englund and Cai [46] for Hawaii; Jabłońska et al. [47] for Poland; Klotz et al. [48] for Germany). Thus, some populations might have resulted from introduction into the wild from aquarium stocks.

*Neocaridina davidi* in Taiwan can be divided into three lineages (ND-ND3, Fig. 5a). Lineage ND2 was only distributed north of the Miaoli Plateaus, lineage ND3 was mostly distributed in northeastern Taiwan, and lineage ND1 was widely distributed. The results of the S-DIVA revealed that the ancestral populations were distributed north of the Formosa Bank and in northeastern Taiwan. Moreover, our study found that some populations did not have private haplotypes, and these populations were not distributed in ancestral areas, excluding population FG (Table 1; Figs. 1 and 5a). In addition, the shared haplotypes of the other three species were only distributed in the neighbour populations, and the shared haplotypes of *N. davidi*, excluding D9 and D10, were also distributed in the neighbour populations (Table 1). Thus, we suggest that the distribution of the widespread haplotype and the discontinuous distribution might have resulted from introductions to the wild from aquarium stocks, and the human-caused transformations among the wild population were rarer than the introductions of individuals to the wild from aquarium stocks.

In conclusion, our study considered that the ancestral populations of *N. davidi* were distributed north of the Formosa Bank and in northeastern Taiwan (Fig. 5a) and then isolated and divergent by the Central Range and Miaoli Plateau. Finally, the ND1-ND3 lineages were restricted to northeastern Taiwan (E region), north of the Miaoli Plateau (A region), south of the Miaoli Plateau and north of the Formosa Bank (B region). We suggested that the populations in regions C and D might have resulted from the introduction from aquarium stocks to the wild by humans (Fig. 5a).

## Conclusions

This study found that there were four *Neocaridina* species in Taiwan and that they originated through four colonization events. There were five phylogeographic breaks in Taiwan: the Central Range, Taoyuan Plateau, Miaoli Plateau, Formosa Bank and Kaoping foreland basins. This study found that the population sizes of these four species all displayed declines (Fig. 4). In the census programme of the *Neocaridina* species in Taiwan, we found that the *Neocaridina* species is rare or has disappeared in many rivers. Thus, the results of the present study provide information to conservation management agencies about the patterns of genetic diversity and the structure of the *Neocaridina* species in Taiwan. However, this study did not determine the systematics of the genus *Neocaridina* in East Asia*.* The results of the present study provide information on the phylogeography of East Asia and the history of the genus *Neocaridina*. In future studies, we need more sampling and more genetic characters.

## Methods

### Population sampling and molecular methods

A total of 479 specimens of *Neocaridina* species were collected from 35 localities in Taiwan, which almost covers all rivers within the island (Fig. 1; Table 1). *Neocaridina* species are not endangered or protected species, and the fieldwork was conducted in accordance with the guidelines established by the National Museum of Marine Biology and Aquarium in Taiwan. All specimens were stored in the laboratory of Chiao-Chuan Han, National Museum of Marine Biology and Aquarium*.* The shrimp were collected from field sites with seines and fatally anaesthetized with MS-222 (Sigma, St. Louis, MO). The samples were fixed and stored in 100% ethanol. The genomic DNA was extracted from the muscle tissue using the Genomic DNA Purification Kit (Gentra Systems, Valencia, CA, USA). The partial COI gene was amplified by polymerase chain reaction (PCR) using the primers LCO1490 (5′-GGTCAACAAATCATAAAGATATTGG-3′) and HCO2198 (5′-TAAACTTCAGGGTGACCAAAAAATCA-3′) [49]. Each 50 μl PCR mixture contained 5 ng of template DNA, 5 μl of 10x reaction buffer, 4 μl of dNTP mix (10 mM), 5 pmol of each primer and 2 U of Taq polymerase (TaKaRa, Taq polymerase). The PCR was programmed on an MJ Thermal Cycler for one cycle of denaturation at 94 °C for 3 min, 40 cycles of denaturation at 94 °C for 30 s, annealing at 55 °C for 30 s and extension at 72 °C for 1 min 30 s, followed by a 72 °C extension for 10 min and 4 °C for storage. The purified PCR products were sequenced using an ABI 377 automated sequencer (Applied Biosystems, Foster City, CA, USA). The chromatograms were checked with the CHROMAS software (Technelysium), and the sequences were manually edited using BIOEDIT 6.0.7 [50]. All new sequence data were submitted to GenBank (MG734216-MG734300). Moreover, to identify the *Neocaridina* species and find the origins of the genus *Neocaridina* in Taiwan, our study also downloaded the sequences in Shih et al. [2] from GenBank (AB300177–90 and LC324764–79). In addition, our study also sampled some specimens in the Yangtze River (population CJ) and Hanjiang River (population HJ) in mainland China (Fig. 1).

### Sequence alignment and phylogenetic inferences

The nucleotide sequences were aligned in Clustal X 1.81 [51]. The selection of the best-fit nucleotide substitution models was performed using the Bayesian information criterion (BIC) in jModelTest 2.0 [52]. The most appropriate nucleotide substitution model was HKY + I + G (Hasegawa-Kishino-Yano). The phylogenetic relationships among all haplotypes were inferred using Bayesian inference (BI) and maximum likelihood (ML) in BEAST 1.8.0 [53] and MEGA 6 [54]. For the BEAST analysis, a stick clock model with a Bayesian Skyline tree was used. We ran 10^6^ generations. The burn-in and plots for each analysis were visualized using Tracer v1.6 [55] to determine whether the convergence and suitable effective sample sizes were achieved for all parameters. The TREEANNOTATOR in the BEAST package was used to summarize the tree data, and the tree was viewed using FigTree v1.3 [56]. For ML analysis, bootstrapping was performed with 1000 replications. In addition, the time to the most recent common ancestor (T_MRCA_) was also calculated using the software package BEAST. The substitution rates of 2.33% per million years for terrestrial *Sesarma* [2, 5, 30] and 1.1% per million years for Decapoda [31] were used.

### Population genetic diversity

The intra-population genetic diversity levels were estimated using haplotype diversity (*h*) [57] and nucleotide diversity (θ_π_ and θ_ω_) indices [58] in DnaSP v5 [59]. The current genetic diversity estimates (θ_π_) were based on the pairwise differences between the sequences, and the historical diversity estimates (θ_ω_) were based on the number of segregating sites among the sequences. Comparing the estimates generated by these two indices provided insight into the population dynamics over recent evolutionary history [60]. The existence of a phylogeographic structure was examined following the method of Pons and Petit [61] by calculating two genetic differentiation indices (*G*_ST_ and *N*_ST_) in DnaSP.

### Population history

To determine the potential diversification scenarios, a statistical dispersal-vicariance analysis (S-DIVA), which complements DIVA, was employed to determine the statistical support for the ancestral range reconstructions [62]. The tree file formats were generated using the program BEAST. The range information was defined using the ichthyofaunal classification and phylogeographic studies [10, 32]. The analysis was performed using the ‘maxareas = 2 to 5’ option (see RESULTS: Population diversity of Taiwan species; Fig. 5).

In addition, the demographic histories were reconstructed using three different approaches. First, we performed Tajima’s D and Fu’s F_S_ neutrality tests [63, 64] in DnaSP. Under a population expansion model, the significant negative values of Tajima’s D and Fu’s F_S_ were expected. Second, the mismatch distribution [65] was estimated under the assumption of a sudden expansion model as implemented in Arlequin version 3.5 [66]. The sum of the squared deviations (SSD) between the observed and expected mismatch distributions and the raggedness index (Rg) were used as test statistics with the 1000 bootstrap replicates. In the third approach, we reconstructed the historical demography using the coalescent-based Bayesian skyline plot approach (BSP) implemented in software package BEAST.

To reconstruct the unknown history of divergence, we performed approximated Bayesian computations (ABC) using DIYABC v.2.0 [67]. The DIYABC program enabled the comparison of the different historical scenarios involving population divergence, admixture and population size changes and subsequently inferred the demographic and historical parameters under the best-supported scenario. The reference table was built with 1000,000 simulated data sets per scenario using the following summary statistics: one-sample statistics for the number of haplotypes, Tajima’s D, the mean number of pairwise differences, the variance in the pairwise differences, and the number of segregating sites; two-sample statistics for the mean of the within-sample pairwise differences, the mean of the between-sample pairwise differences, the number of segregating sites and *F*_ST_ between samples. The uniform priors for all scenarios were used, and no constraints on population sizes and coalescent times were given. All the scenarios were compared using direct (D) and logistic regression (L) approaches, and parameter estimation was performed only for the scenarios with the highest posterior probability.

## Data Availability

All mtDNA sequences generated in this study have been deposited in GenBank under accession numbers: MG734216-MG734300.

## Abbreviations

ABC: Approximate Bayesian Computations
BI: Bayesian inference
COI: Cytochrome c oxidase subunit I
ML tree: Maximum- Likelihood tree
Rg index: the raggedness index
S-DIVA analysis: Statistical Dispersal-Vicariance Analysis
SSD: the sum of square deviations
